# Adapted tolerance to virus infections in four geographically distinct *Varroa destructor*-resistant honeybee populations

**DOI:** 10.1038/s41598-021-91686-2

**Published:** 2021-06-11

**Authors:** Barbara Locke, Srinivas Thaduri, Jörg G. Stephan, Matthew Low, Tjeerd Blacquière, Bjørn Dahle, Yves Le Conte, Peter Neumann, Joachim R. de Miranda

**Affiliations:** 1 Department of Ecology, Swedish Species Information Centre, Swedish University of Agricultural Sciences, Uppsala, Sweden; 2 Bio-Interaction and Plant Health, Wageningen University and Research, Wageningen, The Netherlands; 3 Department of Animal and Aquacultural Sciences, Norwegian University of Sciences, Kløfta, Ås, Norway; 4 Abeilles et Environnement, French National Institute for Agricultural Research, Avignon, France; 5 Vetsuisse Faculty, University of Bern, Bern, Switzerland; 6 Agroscope, Swiss Bee Research Center, Bern, Switzerland

**Keywords:** Entomology, Evolutionary ecology, Molecular ecology, Coevolution, Virus-host interactions

## Abstract

The ectoparasitic mite, *Varroa destructor*, is unarguably the leading cause of honeybee (*Apis mellifera*) mortality worldwide through its role as a vector for lethal viruses, in particular, strains of the *Deformed wing virus* (DWV) and *Acute bee paralysis virus* (ABPV) complexes. Several honeybee populations across Europe have well-documented adaptations of mite-resistant traits but little is known about host adaptations towards the virus infections vectored by the mite. The aim of this study was to assess and compare the possible contribution of adapted virus tolerance and/or resistance to the enhanced survival of four well-documented mite-resistant honeybee populations from Norway, Sweden, The Netherlands and France, in relation to unselected mite-susceptible honeybees. Caged adult bees and laboratory reared larvae, from colonies of these four populations, were inoculated with DWV and ABPV in a series of feeding infection experiments, while control groups received virus-free food. Virus infections were monitored using RT-qPCR assays in individuals sampled over a time course. In both adults and larvae the DWV and ABPV infection dynamics were nearly identical in all groups, but all mite-resistant honeybee populations had significantly higher survival rates compared to the mite-susceptible honeybees. These results suggest that adapted virus tolerance is an important component of survival mechanisms.

## Introduction

The ectoparasitic mite, *Varroa destructor*, is inarguably the leading cause of honeybee (*Apis mellifera*) mortality world-wide, practically exterminating wild colonies and severely affecting the management and profitability of beekeeping in the wake of its global spread during the 1980’s and 1990’s^1^. The damage this parasite causes to its new host by feeding on adults and brood is amplified by the multiple viruses it carries and transmits^2–4^. Two virus-complexes in particular are transmitted highly efficiently by varroa mites with devastating consequences^5^: the *Deformed wing virus* (DWV) complex, including major strains DWV-A, DWV-B and DWV-C^6^; and the *Acute bee paralysis virus* (ABPV) complex, including major strains ABPV, *Kashmir bee virus* (KBV) and *Israeli acute paralysis virus* (IAPV)^7^. Both DWV and ABPV are single-stranded RNA viruses that infect all stages of honeybee development^7, 8^. In the absence of varroa mites, they are maintained in the colony at low levels as innocuous infections through horizontal and vertical transmission routes^9–14^. DWV is the most common and wide spread virus^2^. Symptoms are almost exclusively associated with varroa-mediated transmission when the mite feeds on the bee during the pupal developmental stages^8, 15^ causing severe wing deformities that result in flightless adult bees that die shortly after emerging^8^. ABPV symptoms are characterized mostly by severe pupal mortality and by trembling, paralysis and behavioural inadequacies of adult bees at elevated titres^7^.

A natural mite population in an infested honeybee colony can grow exponentially, rapidly leading to a DWV and/or ABPV epidemic that ultimately precipitates the death of the colony typically within a few years^5, 12, 16^. To avoid virus epidemics and thus colony death, mite population control strategies are essential in apiculture in almost all parts of the world where the mite exists^16^. However, there are several extraordinary honeybee populations in Europe and North America that have been documented to survive for extended periods without mite control measures and without the harmful effects typically associated with varroa mite infestation^17^. These populations have all been exposed to the selection pressure of long-term uncontrolled mite infestation and in response have adapted a repertoire of mite-resistant traits that limit the mite population growth rate^18–21^. These traits subsequently support colony survival likely by reducing the transmission potential for lethal virus epidemics. However, despite having acquired mite-resistant traits, these populations can still experience and survive with occasional high mite infestation levels. Suggesting that perhaps other adapted survival mechanisms are contributing to the long-term survival success of these populations such as host resistance or tolerance to the actual mortality inducing virus infections.

In host–parasite interactions, host tolerance is defined as the ability to reduce the effect of the parasite, while host resistance is the ability to reduce the fitness of the parasite^22^. Recently, we have shown that individuals from the naturally adapted mite-resistant honeybee population on Gotland, Sweden, survive with higher thresholds of DWV and ABPV infections before bee health is compromised, relative to mite-susceptible unselected honeybees^23^. This suggests that host tolerance, rather than resistance, to virus infections is an important component of the naturally adapted survival mechanisms of the Gotland mite-resistant population, in addition to their adapted mite-resistant traits. At the colony level, the Gotland honeybee population also appear to have adapted resistance to other virus infection not directly transmitted by varroa mites but nevertheless harm honeybee health and reduce long-term survival^24, 25^.

The aim of this study was to assess the possible contribution of adapted virus tolerance and/or resistance to the enhanced survival of three other well-documented mite-resistant honeybee populations from Norway^20^, The Netherlands^21^ and France^17, 19, 26^, while comparing them with the Gotland population in Sweden^17, 18, 23^ and a non-selected mite-susceptible local honeybee population. This was done by comparing how experimental APBV and DWV oral infections differentially affect the larvae and adult bees from these honeybee population, through both a virus infection time-course and adult bee mortality rates. Virus susceptibility was determined by comparing the virus titres of virus-inoculated bees relative to both the pre-experiment background virus titres and the natural infection development in uninoculated bees across the time-course. Adult bee mortality over time was also recorded, as well as the virus titres in dead bees.

## Materials and methods

### Origin of honeybee colonies

The origin, management and varroa-resistance characteristics of the four varroa-resistant populations has been abundantly described^18–21, 26, 27^, and recently summarised in a review^17^. Briefly, the populations have evolved independently without mite control since 1994 (Avignon, France)^26^, 1999 (Gotland, Sweden)^27^, 2001 (Oslo, Norway)^20^ and 2005 (Tiengemeten, The Netherlands)^21^. During summer 2016, twelve queens from each of the four mite-resistant populations were produced and mated in their geographic locations of origin and were transported by surface courier to Sweden according to EU-legislation guidelines for animal transport. Twelve queens from a local mite-susceptible population were similarly produced and mated, to be used in this study as a control group. These queens originated from an unselected population near Uppsala that requires regular varroa mite control interventions by beekeepers to avoid colony death. Sixty host colonies, each in a single full-size hive body containing 4 frames brood, 1 frame pollen, 2 frames honey and 3 frames wax foundation, were acquired from four local beekeepers (15 colonies from each beekeeper) and placed in four apiaries (one apiary per beekeeper) at the Lövsta Research Station at the Swedish University of Agricultural Sciences in Uppsala, Sweden. The four apiaries were between 500 and 1000 m separated from each other. On July 12, 2016, in each of the four apiaries, three queens from each of the five populations (Control, Dutch, French, Norwegian and Swedish) were introduced in the fifteen colonies. All colonies were fed with a commercial 66% w/w sugar solution with a 2/1/1 ratio of sucrose/fructose/glucose (Bifor®, Nordic Sugar A/S, Copenhagen, Denmark) to encourage queen acceptance. On August 25, 2016 each colony was treated against varroa with two strips of tau-fluvalinate (Apistan®, Vita Europe, UK) for six weeks, following the manufacturers recommended procedures. No further varroa treatment was applied until the experiments described here began the following summer in 2017, by which time all individuals in each colony were the offspring of the introduced experimental and control queens. The colonies used in these experiments were selected randomly from all four apiaries. Samples of ~ 300 adult bees were taken from each colony at bi-monthly intervals during 2017 to determine the phoretic varroa infestation rates, using soapy water mite washes^28^, with the rate for August 2017 (when the larvae and adults for the experiments were collected) used in the analyses (Supplementary Fig. 1a).

### Preparation and optimization of virus material

The DWV and ABPV inocula used in each oral infection experiment was prepared previously^23^ in accordance with standard pupal virus propagation procedures^13^. In brief, the inocula were prepared by propagating reference stocks of DWV-A and ABPV each in fifty white eyed pupae from varroa-free colonies^23^. Each pupae was injected with 1 micro litre of a 1/10,000 dilution of purified concentrated virus stock (equivalent to about 10^3^ virus genome copies/bee)^13^. From these 50 pupae, a clarified crude extract was made by homogenizing the pupae in a blender with 10 mL 0.5 M Phosphate Buffer, pH 8.0 (DWV) or 10 mL 0.01 M Phosphate Buffer, pH 7.0 (ABPV),and stored in 50 μl aliquots at – 80 °C^13^. These crude extracts were used for the virus infection experiments. The virological composition of the propagated virus stocks has been described previously^19^ and was determined using RT-qPCR assays for seven common bee viruses that can be propagated through injection^13^: DWV-A, DWV-B, ABPV, IAPV, KBV, *sacbrood virus* (SBV) and *black queen cell virus* (BQCV).

The infectivity of the crude extracts was tested previously in optimization experiments^23^ to identify the optimum dose for experimentation that did not cause larvae or adult bee mortality before 96 h post inoculation (hpi). This dose selection criteria was used so that early (non-lethal) virus infectivity dynamics could be studied, as well as possible subsequent differential mortality between mite-resistant and mite-susceptible bees. The optimum single inoculation dose for larvae was determined to be ~ 1.5 × 10^8^ and ~ 6.0 × 10^8^ DWV genome equivalents for larvae and adults respectively, with the corresponding figures for ABPV inoculation ~ 5.4 × 10^7^ and ~ 2.1 × 10^8^ ABPV genome equivalents, as determined by RT-qPCR analysis of the crude extracts. These levels are consistent with previous estimates of the infectious doses for these viruses^13, 15, 23, 29, 30^.

### Experimental design

The infection experiments were conducted separately on newly emerged adult bees and on newly hatched larvae. Each infection experiment consisted a single infection time-course for bees from four different colonies from each population. With a few exceptions, the same colonies were used for both the larval and adult experiments. Each infection trial consisted of one cohort of DWV-inoculated bees, one cohort of ABPV-inoculated bees and one cohort of non-inoculated control bees. From each cohort of bees in each infection trial, adult bees and larvae were sampled at 0, 6, 24, 48, and 72 h post inoculation (hpi) representing the time-course. The inoculation strategy consisted of feeding bees with a single infectious dose for a short period followed by non-contaminated food for the remainder of the time course, in order to ensure that any increase in virus titres through the time course represented a newly established infection rather than a passive accumulation of virus inoculum. The non-inoculated cohorts received food containing crude extract from non-inoculated pupae.

### In-vitro larval infection experiments

The larval infection experiments were conducted on newly hatched larvae from 4 colonies from each of the four mite-resistant populations (**N**orwegian, **S**wedish, **D**utch, **F**rench) and the mite-susceptible **C**ontrol population. Larvae of similar age were obtained by confining the queens of the experimental colonies to a single frame for 24 h for egg-laying. First instar larvae (between 24 and 36 h old) were transferred into individual wells of 48-well tissue culture plates (Falcon™ Polystyrene Microplates) following standard larval rearing procedures^23, 31, 32^. The larvae were pre-incubated for 24 h at 35 °C with a relative humidity of 96%, after which all dead and excess larvae were removed, such that 48 living larvae were retained for the infection experiment. The viable larvae were then fed with larval food. For the larvae cohorts to be inoculated with virus, the larval food was mixed with the optimum single infectious dose of DWV or ABPV, as determined above. The larvae were fed daily according to established protocols^32^. At each hpi-sampling point during the experimental time-course (see above), four live larvae from each infection cohort were collected in microcentrifuge tubes and stored at − 20 °C until further analysis^33^.

### Adult bee cage infection experiments

The adult infection experiments were conducted on newly hatched adult bees from (generally) the same 4 colonies used for the larvae infection experiments from each of the five populations. The bees were hatched on caged frames inside an incubator at a constant 35 °C temperature and 96% relative humidity^34^. The level of varroa infestation of each frame was assessed on a four-point ordered scale (0–4 for none, low, medium and high) based on the number of mites encountered in the cage during the emergence of the adults (Supplementary Fig. 1b). For each inoculation trial, cohorts of fifty newly emerged adults from each colony were placed in separate Lyson queen cages (Łyson, Klecza Dolna, Poland), and fed the optimum DWV and ABPV inoculation dose (as described above) in 2 mL Bifor® (Nordic Sugar A/S, Copenhagen, Denmark) over a 24-h period, with uninoculated bees receiving just Bifor®. After inoculation, all cohorts of bees were fed unadulterated Bifor® ad libitum for the remainder of the time-course. On each sampling occasion (see above) and for each infection cohort, five live bees were sampled. To study the survival rate of each population to virus infections, all dead bees were also counted and removed at each sampling time point. The experiments continued for six days (144 hpi), after which the number of dead and surviving bees were counted. All the sampled live and dead bees were stored at − 20 °C until further analysis.

### Sample processing and RT-qPCR assays

Each experimental time-course sample, containing either 4 larvae or 5 adult bees, was placed in a mesh bag and ground to powder using liquid nitrogen and a pestle. A primary homogenate was produced by adding 200 μl/bee sterile water to each ground sample and mixing vigorously^33^. Total RNA was extracted from 100 μl of this homogenate by a QiaCube robot following the RNAeasy protocol for plants (Qiagen). The RNA was eluted in 50-μl RNase-free water, the RNA concentration was estimated by NanoDrop and the purified RNA was stored at − 80 °C until further processing.

The amounts of DWV and ABPV RNA, as well as RP49 mRNA (a honeybee internal reference gene commonly used for normalizing between-sample differences in RNA quantity and quality^13^) were determined using reverse transcription quantitative PCR (RT-qPCR), using the iScript One Step RT-PCR kit (Bio-Rad) with SYBR Green as the detection chemistry and the Bio-Rad CFX connect thermocycler. The reactions were performed in 20 μl volumes containing 0.2 μM of the forward and the reverse primers, 3 μl RNA, 10 μl SYBR Green RTmix and 0.4 μl of iScript reverse transcriptase, with the following cycling profile: 10 min at 50 °C for cDNA synthesis, 5 min at 95 °C for inactivation of the reverse transcriptase following 40 cycles of 10 s. at 95 °C for denaturation and 30 s. at 58 °C for annealing/extension and data collection. Amplification was followed immediately by a Melting Curve analysis to confirm the identity of the amplification products, by incubating at 60 s: 95 °C, 60 s 65 °C and fluorescence reading at 0.5 °C increments between 65 and 95 °C. Included in each qPCR run was a ten-fold dilution series of known amounts of each target, for absolute quantification. All assays were run in duplicate, with the average Cq value retained for analysis. The qPCR data were first screened for the presence of secondary RT-PCR products through visual inspection of the Melting Curve (MC) analyses. After the visual inspection, the average Cq values were converted to Standard Quantity (SQ) values through use of the external calibration curves established by the ten-fold dilution series for each target. These data were then multiplied by the various dilution factors throughout the methodology to estimate the copy number of each target per bee. The DWV and ABPV values for each sample were then normalized using their corresponding RP49 values, to correct for sample-specific differences in the quality and quantity of RNA^35, 36^.

### Statistical analyses

The normalized virus titres per individual bee were log_10_ transformed and analysed with General Linear Mixed Models using the R software^37^. The titres of either of the two viruses (DWV, ABPV) as recorded for the infection experiments for either of the two life stages (larvae, adults) were used as the response variables, analysed separately. In each of these four separate analyses the models (function lmer within the lmerTest package^38^) tested the degree to which the virus titres depended on the **Population** of origin, the **Inoculation** treatment (DWV, ABPV, no virus) or the phoretic **Varroa** infestation rate of the colonies providing the experimental bees, and their interactions. The apiary location of the colonies and the different post-inoculation sampling time-points during the time-courses were included as Gaussian random effects to account for, respectively, any apiary-specific variability between the colonies used and the repeated measure structure associated with sampling the same group of bees progressively during the time-course. The distribution of the residuals and the homogeneity of variances was checked visually to confirm compliance with the assumptions for linear models using a Gaussian-distributed response variable^39^. Pairwise comparisons (using the function glht and cld from the mulcomp package^40^) among all combinations of **Population** and **Inoculation** treatment were calculated from these initial models. In order to evaluate the importance of each of these predictors and their interaction, non-significant terms were removed in a backwards model selection (using the step function in the lmerTest package) until the minimal adequate model was obtained.

To examine differences in individual bee mortality probability between varroa-susceptible and varroa-resistant populations, we implemented a time-to-event (or ‘survival’) analysis using a Cox proportional hazards regression model^41^. Here we estimated the probability that an individual bee died relative to time post-inoculation. These probabilities were compared between the different populations, in particular with pairwise comparisons between the varroa-susceptible Control and each of the Dutch, French, Norwegian and Swedish varroa-resistant populations. The analyses were run separately for DWV-inoculated, ABPV-inoculated and non-inoculated adult bees. For each of these three virus inoculation experiments, we followed the fate of 600 individual bees (30 bees from each colony × 4 replicate colonies per population × 5 population) and recorded their survival or mortality at 6, 24, 48, 72, 96 and 144 h post-inoculation (a total of 3600 potential individual bees observations). Because the colonies were located at four different apiaries, with different historical and environmental backgrounds (see “Origin of honeybee colonies”), we included ‘location’ as a random effect with a so-called ‘frailty model’^42^. Without this random effect, the Schoenfeld analysis of the residuals^43^ indicated some violation of the proportional hazards assumptions. For all Cox Proportional Hazard analyses we included two covariates to explain differences in mortality: the population origin (i.e. Control, Dutch, French, Norwegian and Swedish) and the colony-level phoretic varroa infestation rate (mites per adult bee) for each colony during August 2017, when the experiments took place, to control for any confounding effects that different varroa mite loads would contribute to individual bee mortality. The models converged readily (11, 12 and 14 outer iterations; 28, 31 and 35 Newton–Raphson iterations) and could explain the data with a very high degree of confidence, as assessed by a Likelihood ratio test (Supplementary Table 1). The analyses were conducted in R^37^ using the ‘coxme’ package^44^.

## Results

### Virus infection time courses

The virus infection time courses for the two viruses were very similar to those reported previously^19^. A graphical summary of the raw data is shown in Supplementary Fig. 2. For the DWV infection experiments in larvae there was large increase in DWV titre between the pre-inoculation and the first post-inoculation time-point (at 6 hpi), followed by slight increases for the remainder of the time-course, in all five populations. There were generally no DWV titre increases in either the non-inoculated control series or the ABPV-inoculated series, except for the French population and, to a lesser degree, the Dutch population. The DWV infection experiments in adults were compromised by the very high background DWV titres in the populations, similar to what we observed previously^19^, making it impossible to demonstrate successful infection either through comparing pre- and post-inoculation samples or through titre increases with time, since such increases were observed equally in all populations for the DWV-inoculated, the ABPV-inoculated and the non-inoculated series. It is only through the effect of DWV inoculation on adult mortality (see later) that we know that the inoculum actually had an effect on the bees. For the ABPV infection time-course experiments there is clearer evidence of a slow progressive increase in ABPV titres, in both larvae and adult bees, and for all populations, suggesting that the inoculation resulted in an active infection. There was no great difference in the background ABPV titres between the DWV-inoculated and non-inoculated individuals for either the larval or adult infection experiment. For the adult experiment there was a slight increase in these background ABPV titres over time, whereas for the larval experiment there was not.

### Virus susceptibility

**Figure 1:**
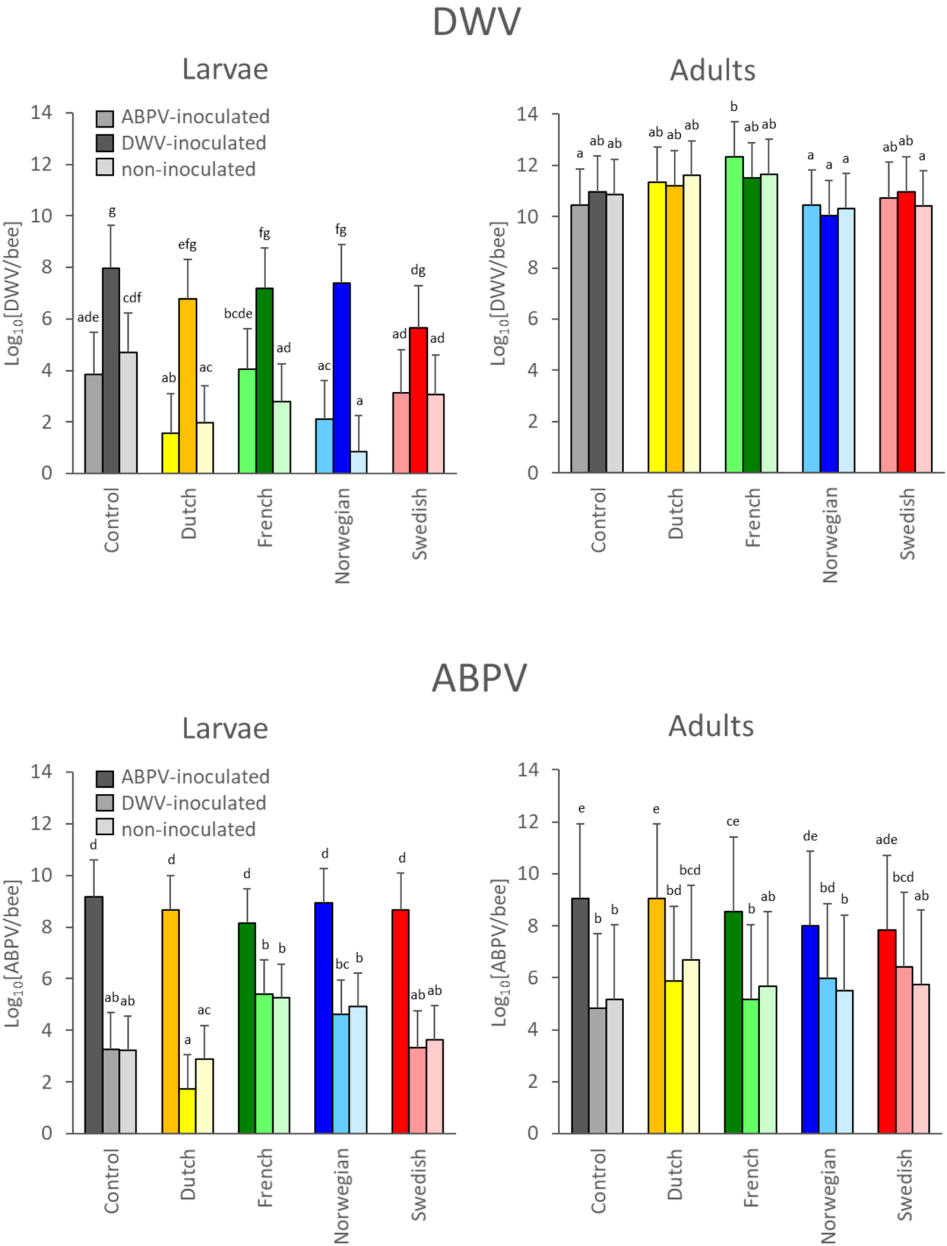
Graphical representation of the DWV (top panels) and ABPV (bottom panels) titres pooled across the entire larval (left panels) and adult (right panels) infection time-course experiments for the virus-susceptible Control population (grey) and the Dutch (yellow), French (green), Norwegian (blue) and Swedish (red) varroa-resistant populations. The dark bars concern the data where the PCR assay detects the inoculated virus. The other bars concern the data where the PCR assay detects background virus, either in the non-inoculated control series (light bars) or the series inoculated with the alternate virus (medium bars). The mean values were estimated using Generalized Linear Mixed Models with time and colony as a random factor and colony-level phoretic varroa infestation rate as an explanatory factor. The error bars represent the 95% confidence interval on the estimate. The letters are used to identify differences between population and inoculation combinations within each virus-life stage combination.

Although both inoculated and background virus titres tended to increase slightly over time in both the larval and adult experiments and for all populations, often enough to suspect that these represented active infections, these increases were not large enough with respect to the replicate error variance to be significant. For the remainder of the analyses therefore the values from the time-course were pooled, effectively treating time post-inoculation as a random factor in the GLMM analyses. This meant that the data from the entire time-course were compressed into a single value, which can be taken as a measure of the overall susceptibility of the population to DWV or ABPV infection over the entire time-course, as well as the susceptibility to background DWV or ABPV infections due to inoculation with the alternate virus (Fig. 1; Supplementary Table 1). These estimates include a correction for the independent effect of the colony-level varroa infestation rates on the susceptibility to oral DWV or ABPV inoculation (Table 1; Supplementary Fig. 3).

**Table 1:** Analysis-of-deviance tables (Type III test) for the four linear mixed models investigating the effect of honeybee population (Control, Dutch, French, Norwegian, Swedish) and inoculation treatment (DWV, ABPV, non-inoculated) on the titres of DWV and ABPV in larvae and adults.

Virus	Life stage	Variable	Variation	DF^v^; DF_e_	F-value	*P*
DWV	Larvae	Population	25.47	4; 219.31	4.57	0.001
		Virus	452.22	2; 224.02	81.22	< 0.001
		*Population:Virus*	*12.83*	*8; 224.66*	*2.30*	*0.022*
		varroa	60.23	1; 227.23	10.82	0.001
DWV	Adult	Population	17.64	4; 260.36	8.32	< 0.001
		*Virus*	*0.37*	*2; 259.86*	*0.17*	*0.842*
		*Population:Virus*	*1.82*	*8; 248.68*	*0.85*	*0.560*
		varroa	165.42	1; 261.64	77.99	< 0.001
ABPV	Larvae	Population	25.01	4; 225.26	7.48	< 0.001
		Virus	608.12	2; 224.48	181.86	< 0.001
		Population:Virus	12.18	8; 224.25	3.64	0.001
		varroa	41.60	1; 227.05	12.44	0.001
ABPV	Adult	*Population*	*5.61*	*4; 233.11*	*1.58*	*0.182*
		Virus	228.15	2; 262.22	63.34	< 0.001
		*Population:Virus*	*6.31*	*8; 249.37*	*1.82*	*0.074*
		varroa	29.45	1; 100.69	8.18	0.005

Non-significant terms (italicized) were removed stepwise, starting with the Population:Virus interaction term, until the final minimal adequate model was obtained. Indicated are the **Virus** studied; the bee **Life stage** investigated in the inoculation time-course; the explanatory **Variable** analysed (**Virus** inoculation treatment; honeybee **Population** of origin; their **Population:Virus** interaction and the effect of the colony-level phoretic **varroa** infestation rate for each colony (see Supplementary Fig. 3 for the direction of the effect)); the variation for each variable; the Degrees of Freedom associated with the target variable (**DF**^**v**^) and the combined error variance (**DF**_**e**_), derived from the replicate colonies and the multiple post-inoculation sampling time-points; the **F-value** representing the ratio between the target MS and the error variance, and the probability (***P***) of obtaining this F-value by chance, given the DF^v^ and DF_e_. Significance was set at *p* < 0.01, with non-significant items shown in *italics.*

For DWV titres as the response variable, there is a clear difference between the five populations in susceptibility of the larvae to oral inoculation with DWV, with the varroa-susceptible Control population colonies considerably more susceptible than the varroa-resistant Dutch, French, Norwegian and (especially) Swedish populations (Fig. 1; Table 1). There is also divergence in background DWV susceptibility between the five populations, in the ABPV-inoculated or non-inoculated larvae, with the Dutch and Norwegian populations having noticeably lower background DWV infection than the Swedish, French and Control populations. However, for each population there was no significant difference in DWV susceptibility between ABPV-inoculated or non-inoculated larvae, as we also found previously^23^. As indicated above, the adult infection experiment was dominated by the extremely high background DWV levels, making it impossible to detect any additional increase in DWV titre due to oral inoculation. Consequently, the DWV titres for the DWV-inoculated, ABPV-inoculated and non-inoculated bees were similar within each population, with no effect of the different virus inoculation treatments (Fig. 1; Table 1). The only significant difference is between the different populations, with the highest DWV titres in the French bees, followed by the Dutch, Control, Swedish and Norwegian bees (Fig. 1; Table 1).

The general pattern seen for the DWV titres as response variable was repeated with ABPV titres as the response variable, but with some key differences. Again, the inoculation treatment was highly significant, both in larvae and in adults, as shown by the difference between the ABPV titres in the ABPV-inoculated series compared to the DWV-inoculated and non-inoculated series. Again there is no difference in ABPV titres between non-inoculated and DWV-inoculated larvae for each population. For the larval inoculation, there was highly significant difference between the populations in ABPV susceptibility (Table 1), although this effect may be more due to the differences in the background ABPV titres in the DWV-inoculated and non-inoculated larvae than due to the differences between populations in the ABPV-inoculated larvae (Fig. 1). Again, the varroa-susceptible Control population appears to be most susceptible to inoculated ABPV, followed by the Norwegian, Swedish, Dutch and French populations. For the adult bees, the Control population was again most sensitive to ABPV inoculation, with the Dutch, French, Norwegian and Swedish populations progressively less susceptible. The background ABPV titres in the adult bees were relatively similar for all populations, with no difference between non-inoculated and DWV-inoculated bees. The general uniformity between the populations in especially the background ABPV titres, coupled with the relatively high error variance, means that there was no significant difference overall between the populations in ABPV susceptibility (Table 1).

The phoretic varroa infestation rate of the colonies consistently had a highly significant effect on the ABPV and DWV susceptibility of the colonies (Table 1). The relationship between colony-level varroa infestation and DWV or ABPV susceptibility was largely neutral for the larval inoculation experiments (Supplementary Fig. 3), which is not surprising since varroa has no direct interaction with young brood. Any effects of varroa infestation on larval susceptibility therefore has mediated indirectly through colony-level processes. However, the emerging adult bees are directly affected by the colony-level varroa infestation rate (Supplementary Fig. 1a,b), since varroa reproduces on developing pupae^1^. Consequently, relationship between colony-level varroa infestation and virus susceptibility of the adult bees is much more pronounced, with higher infestation rates associated with greater susceptibility to oral virus infection (Supplementary Fig. 3). In all four experiments, the random effects of apiary location (DWV-larvae: *p* < 0.01; DWV-adult, ABPV-larvae: *p* < 0.001; ABPV-adult: *p* < 0.05) and time post infection (DWV-larvae: *p* = 0.06; ABPV-larvae: *p* < 0.05; DWV-adult, ABPV-adult: *p* < 0.001) were relevant.

### Adult mortality

**Figure 2:**
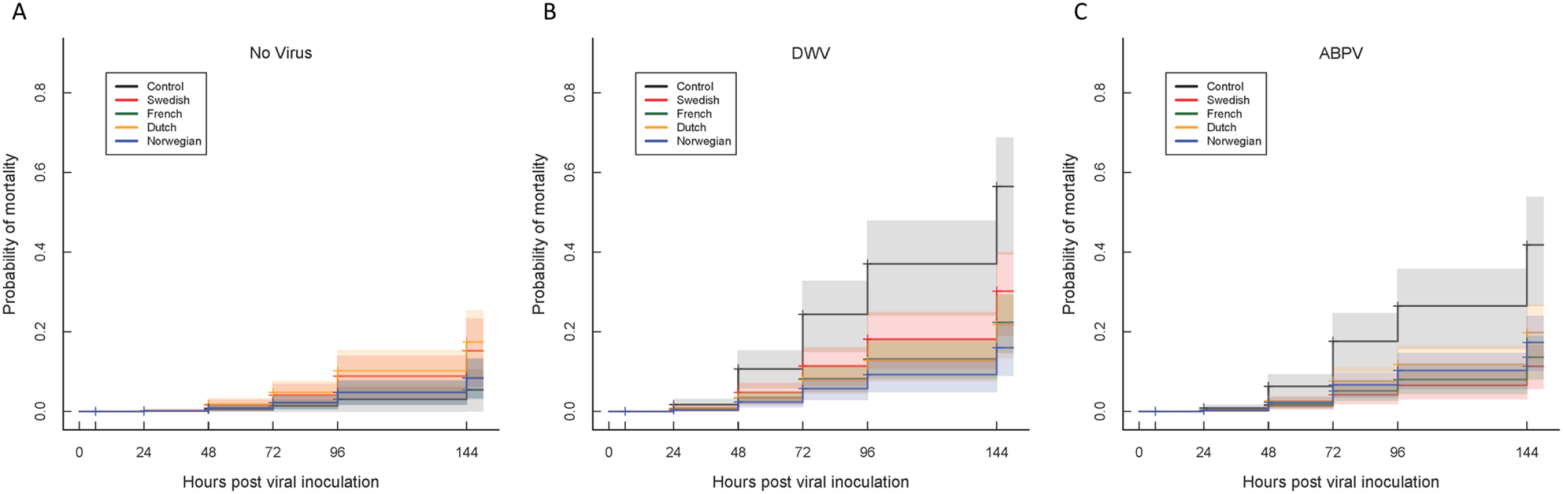
Cox Proportional Hazard curves for the Dutch, French, Norwegian and Swedish varroa-resistant honeybee populations and the varroa-susceptible Control population for the non-inoculated (**A**), DWV-inoculated (**B**) and ABPV-inoculated (**C**) adult bee virus infection experiments. The shaded areas represent the 95% confidence intervals for the proportional hazard lines, based on the data from four replicate honeybee colonies for each population. The effects of varroa infestation and apiary origin of the colonies have been accounted for in the models.

For the adult infection time-course experiment, dead bees were removed daily from the experimental cages for six days (144 h) post-inoculation. This mortality data was analysed with reference to the population of origin and the inoculation treatment, using Cox’s Proportional Hazard analyses. Without any experimental virus inoculation there is no difference between any of the populations in background mortality (Fig. 2A; Table 2), which is an acceptable 15–20% over 6 days. However, after experimental DWV or (especially) ABPV inoculation, adults from the varroa-susceptible Control population are much more likely to die than bees from any of the varroa-resistant target populations (Fig. 2B,C; Table 2). For the DWV-inoculated bees there are also slight differences in mortality between the different varroa-resistant populations, while for the ABPV-inoculated bees this is less evident. Since the colonies used in these experiments had not received any varroa control for about 1 year (see “Materials and methods”), they had varying rates of colony-level varroa infestation at the start of the virus inoculation experiments (Supplementary Fig. 1a), which reflected the subjective assessments of varroa infestation for the emerged adult bees emerged (Supplementary Fig. 1b). Since varroa infestation by itself has an enormous effect on post-emergence adult survival^1, 45^, the colony-level varroa infestation rates were included in the modelling of adult mortality, to neutralize the effect of the differential varroa infestation rates between the colonies and populations (Supplementary Fig. 1) on the primary results, which are the comparisons between the different populations and virus inoculation treatments (Fig. 2; Table 2). However, these analyses revealed some interesting and contradictory results. The most revealing variable in Table 2 is the exponentiated coefficient (**exp**^**coefficient**^) which shows both the size of the effect and the direction, with values < 1.00 indicating a lower mortality due to the factor, and values > 1.00 a higher mortality. As is also shown in Fig. 2, the bees from each of the varroa-resistant populations had a lower mortality than bees from the Control population in both the DWV-inoculation (blue) and ABPV-inoculation (orange) experiments (exponentiated coefficients < 1.00 throughout) while in the absence of experimental virus inoculation (grey), the bees from the Control colonies actually survived better than those from the varroa-resistant control populations (exponentiated coefficients > 1.00 throughout). By the same logic we see that for the non-inoculated bees and ABPV-inoculated bees, colony-level varroa infestation had, as expected, a huge and highly significant negative effect on survival, with very large exponentiated coefficients. However, for DWV-inoculated adults, varroa had a net positive effect on survival (exponentiated coefficient < 1.00), although the results were a little diffuse, as indicated by the modest χ^2^ and associated probability (*p* = 0.02). This result is due to a couple of (Control) colonies with very large varroa infestation rates that significantly overperformed with respect to expectation, together with several low-infestation colonies that underperformed. The interesting result is that the same bees, with the same infestation rates, reacted so very differently depending on whether they were inoculated with DWV, with ABPV or not inoculated at all. Obviously, non-inoculated bees survive much better than DWV-inoculated or ABPV-inoculated bees, with or without varroa (Fig. 2), but the absence of this interactive effect between varroa and oral DWV inoculation may be one more factor explaining why DWV consistently emerges as the primary varroa-associated virus in apiculture^5, 46–48^. The final factor to affect mortality is the apiary origin of the colonies, with a clear and consistent ranking of the four apiaries for all three analyses, with the bees from colonies in the ‘Enköping’ and ‘Rimbo’ apiaries having better survival than expected, and those in the ‘Sigtuna’ and ‘Funbo’ apiaries lower survival than expected, independent of the virus treatment (Table 2).

**Table 2:** Regression **coefficients ± standard errors**, the exponentiated value of these coefficients (**exp**^**coefficient**^), the Chi-squared value (**χ**^2^), degrees of freedom (**DF**) and the associated probability of obtaining these χ^2^ values by chance (***P***) from Cox proportional hazards models describing the probability of mortality for individual bees after being inoculated (**Inoculum**) with DWV or ABPV, as well as for non-inoculated bees (none).

Inoculum	Factor	Coefficient ± SE	exp^Coefficient^	χ^2^	DF	*P*
DWV	Dutch vs Control	− 1.22 ± 0.24	0.30	25.34	1.00	< 0.001
	French vs Control	− 1.18 ± 0.24	0.31	24.42	1.00	< 0.001
	Norwegian vs Control	− 1.57 ± 0.29	0.21	29.52	1.00	< 0.001
	Swedish vs Control	− 0.84 ± 0.25	0.43	10.88	1.00	< 0.001
	Varroa	− 1.58 ± 0.72	0.21	4.82	1.00	0.028
	Frailty (apiary effect)			75.59	2.91	< 0.001
	Enköping		0.22			
	Funbo		1.81			
	Rimbo		0.51			
	Sigtuna		1.46			
ABPV	Dutch vs Control	− 0.89 ± 0.25	0.41	13.19	1.00	< 0.001
	French vs Control	− 1.29 ± 0.27	0.28	23.08	1.00	< 0.001
	Norwegian vs Control	− 1.05 ± 0.28	0.35	13.68	1.00	< 0.001
	Swedish vs Control	− 1.50 ± 0.29	0.22	26.33	1.00	< 0.001
	Varroa	3.19 ± 0.70	24.27	20.76	1.00	< 0.001
	Frailty (apiary effect)			63.92	2.90	< 0.001
	Enköping		0.17			
	Funbo		1.81			
	Rimbo		0.51			
	Sigtuna		1.51			
None	Dutch vs Control	1.25 ± 0.46	3.48	7.23	1.00	0.007
	French vs Control	0.48 ± 0.52	1.62	0.84	1.00	0.360
	Norwegian vs Control	0.46 ± 0.54	1.58	0.71	1.00	0.400
	Swedish vs Control	1.10 ± 0.48	3.01	5.26	1.00	0.022
	Varroa	4.19 ± 1.26	65.94	10.99	1.00	< 0.001
	Frailty (apiary effect)			18.48	2.68	< 0.001
	Enköping		0.21			
	Funbo		1.76			
	Rimbo		0.96			
	Sigtuna		1.07			

The main **Factors** explored for explaining the variation in the data are differences between the varroa-susceptible Control population and each of the four varroa-resistant populations (Dutch, French, Norwegian and Swedish), as well as the effect on adult survival of the phoretic varroa infestation rates of the colonies when the experimental adult bees were sampled (Varroa) and the apiary location of the colonies at SLU’s Lövsta Research Station (**Frailty**), with exponentiated coefficients for each of the four apiaries (‘Enköping’, ‘Funbo’, ‘Rimbo’ and ‘Sigtuna’). The exponentiated coefficients converts the regression coefficients to a proportional scale, where values < 1.00 represent a lower mortality due to the factor, and values > 1.00 a higher mortality. The effects of the four apiaries concern relative internal differences, with the exponentiated coefficients totaling 4.00 in each analysis.

## Discussion

Honeybee colony health and pathogen abundance and prevelence is regulated by a variety of factors including season, geographical location, colony dynamics, pathogen strain and the individual and colony level immune responses^49^. Therefore, laboratory studies, like this one, that remove these confounding factors, are ideal for isolating and exploring the mechanisms involved in the interactions between viruses and bees, especially at individual level. Here we investigated the susceptibility of young larvae and emerging adults from four distinct varroa-resistant honeybee populations to oral inoculation with two major honeybee viruses, DWV and ABPV, relative to those of a varroa-susceptible Control population, as well as the mortality of the adult bees from the experiments. We also looked at the influence of confounding factors on virus susceptibility and adult mortality, such as the colony-level varroa infestation rates and the apiary origins of the colonies supplying the bees for the experiments. The results can be summarised as follows. First, oral inoculation generally elevated virus titres above pre-inoculum titres plus passive acquisition of the inoculum, and increased slightly with time, indicating that the inoculation resulted in infection. The exception is the inoculation of the adult bees with DWV, where the exceptionally high background DWV levels precluded any conclusive evidence of infection. Second, there were clear differences between the different populations in susceptibility to DWV and ABPV infection, either through inoculation and/or as background infections, both before and after correcting for the effects of varroa and apiary origin, with the Control population usually displaying the highest susceptibility. Third, inoculation with one virus generally did not affect the background levels of the other virus relative to the non-inoculated control, for any of the populations or inoculation experiments, similar to what we found previously^23^. Fourth, adult bees from the varroa-susceptible Control population had much higher mortality after oral DWV or ABPV inoculation than bees from any of the four varroa-resistant populations, while there was no major difference between non-inoculated bees from the various populations. Clearly, infection with these viruses is tolerated much better by the varroa-resistant bees than the varroa-susceptible bees, independent of any differences in virus titres. Fifth, varroa infestation and apiary origin have significant effects on virus susceptibility and mortality in adult bees. Colony-level varroa infestation is associated with higher susceptibility of adult bees to both DWV and ABPV infection, and with much higher mortality of both non-inoculated and ABPV-inoculated bees, but not of DWV-inoculated bees. This absence of an independent effect of varroa infestation on adult bee mortality in only DWV-inoculated bees is particularly interesting in light of the many other exceptional features of DWV that are peculiarly adaptive to optimal co-existence with varroa^2, 23–25, 48^, and that have elevated DWV from relative obscurity to global prominence in the wake of varroa^8, 47, 50^. Apiary effects are a well-known source of error in honeybee research, particularly for colony-level experiments. Here we show that such landscape-level differences can also affect individual level laboratory results, serving both as a caution for those contemplating laboratory studies without due regard for the origin of the bees and as a promise for investigating in greater molecular detail the mechanisms linking the influence of the environment on honeybee molecular health^51^.

Another consistent feature of these experiments is the large variation in the background virus levels between the five populations in all experiments, which contributed majorly to the significance of the population effect in the analyses. The only experimental difference between these colonies was the origin of the queens during establishment in 2016, after which the colonies developed according to the local environmental conditions in the apiaries and the colony development characteristics associated with the genotype of the queen. The colonies of the five populations were distributed systematically and evenly among the four apiaries; three colonies of each population to each apiary, with only these colonies present in each apiary. The four apiaries were located near the center of the extensive SLU Agricultural Research Station at Lövsta, several kilometres from any surrounding bee colonies. In the absence of any compelling environmental explanation, the conclusion is that these background virus levels are also a characteristic trait of these colonies and populations, although the significance of this is as yet unclear. The differences are particularly striking for the larvae inoculation experiments. Both DWV and ABPV can be vertically transmitted, from queen through her eggs to the resulting progeny^7, 8, 10, 11, 52^, and from there to the rest of the colony through larval care and social interactions^53, 54^. Any systematic difference between queens in the level of virus infection^52, 55^ and/or the efficiency of vertical transmission would lead to differences in ‘background’ DWV or ABPV infection in young, newly hatched larvae, such as used in these experiments. ABPV is rare in Sweden and Norway^56^, very common in France^4^ and moderately common in The Netherlands^57^, which does not match very well with the relative background ABPV levels in the larvae from these populations. Furthermore, a large survey of virgin and mated queens from southern France showed no ABPV infection of the queen ovaries, in contrast to the high frequency (and levels) of DWV-A, DWV-B and BQCV infection. Therefore, even if the queens did vertically transmit viruses from their geographic origin to the larvae used in these experiments, it would not fully explain all the differences in the larval background virus levels between the populations. It is therefore possible that some of these differences at least are related to the genetic background of the populations, either directly, at individual bee level, or as mediated through colony-level processes that also differ between the populations. For the adult experiments the DWV background levels were so high as to preclude any conclusion of the infectivity of the DWV inoculum in these experiments. Again there are very clear and consistent differences between the populations in the background DWV levels, suggesting a corresponding difference between the populations in background tolerance or resistance to DWV infection. The background ABPV levels in the adult bees were more uniform between the populations, suggesting no such differential tolerance-resistance between the populations for background ABPV infection.

The most significant conclusion from these five major results is that adult bees from naturally varroa-resistant bee populations are much more tolerant to oral DWV or ABPV infection than bees from regular varroa-susceptible control populations, as shown both here and, independently, in our earlier studies^23, 24^. The most likely explanation for this elevated tolerance to virus infections is that the natural adaptation of these populations to uncontrolled varroa infestation included a degree of tolerance to virus infections in addition to their already well established genetically adapted varroa-resistant traits^19, 20^. Recent studies on the naturally adapted mite-resistant honeybee population on Gotland, Sweden, have demonstrated adapted host tolerance to virus infections at both levels of honeybee social and biological organization: the colony^24^ and the individual bee^23^. Apart from this, few studies have explored host-adaptations to virus infections^58, 59^. The populations in this study provide the opportunity to explore possible tolerance or resistance mechanisms that have arose in populations through natural selection having been exposed to long-lasting likely subclinical virus infections inducing a substantial selective pressure. Virus tolerance and resisance could provide a new and exciting avenue for breeding healthier bees. Tolerance is a highly effective mechanistic response to disease^60^. Unlike resistance mechanisms, tolerance adaptations do not inflict harm on the parasite and is therefore expected to fix in the population rather than causing an open-ended antagonistic coevolution, as is the case with resistance evolution^60^. In addition to uncontrolled mite infestations and high virus levels, environmental influences would have also shaped the genetic adaptations in responses to these parasites^61^. The different geographical origins of the populations used in this study, ranging from Scandinavia to South of France, have dramatically different environmental conditions such as the season length, temperature, and floral resources. The possible influence of such environmental factors on the nature of the adaptive process and the consequences for the different varroa-resistant and virus-tolerant traits of these populations is a topic of current and future research.

The varroa infestation rates in the colonies that were used for these experiments significantly increased the probability of adult bee mortality regardless of inoculation with either virus. This is by itself not surprising, since varroa infestation causes a range of physical and physiological effects for both individual bees and the colony. Not only does varroa mite parasitization directly affects the longevity of adult bees^45^, it also acts as both a mechanical and biological vector of DWV and other viruses^53, 62, 63^, significantly enhancing the epidemiological potential and lethality of virus infections^15^. Varroa mite parasitism also comprimises the honeybee host’s immune response to virus infections by suppressing the expression of immune response related genes^64^ and increasing viral titres in the bee, both of which reduce adult bee survivorship and colony fitness^65, 66^.

The interactions between the mites, the viruses and the honeybee molecular antiviral defense mechanisms and immune functions is a subject of considerable current research^67–70^, but also with considerable gaps in knowledge and understanding^71^. The large set of precise samples generated during this study together with the extensive metadata relating to the genetic and environmental background of the colonies provides a rich source of material to address these knowledge gaps from a number of perspectives.

## Supplementary Information

Supplementary Information.

## Data Availability

The datasets generated during and/or analysed during the current study are available from the corresponding author on reasonable request.
