# Supplementary material for: Adapted tolerance to virus infections in four geographically distinct Varroa destructor-resistant honeybee populations
Source: Sci Rep. 2021 Jun 11;11:12359. doi: 10.1038/s41598-021-91686-2 (PMC8196020; doi:10.1038/s41598-021-91686-2)
Supplement: Supplementary file 1 — Supplementary Information. [file 41598_2021_91686_MOESM1_ESM.docx]

**SUPPLEMENTARY FIGURES AND TABLES**


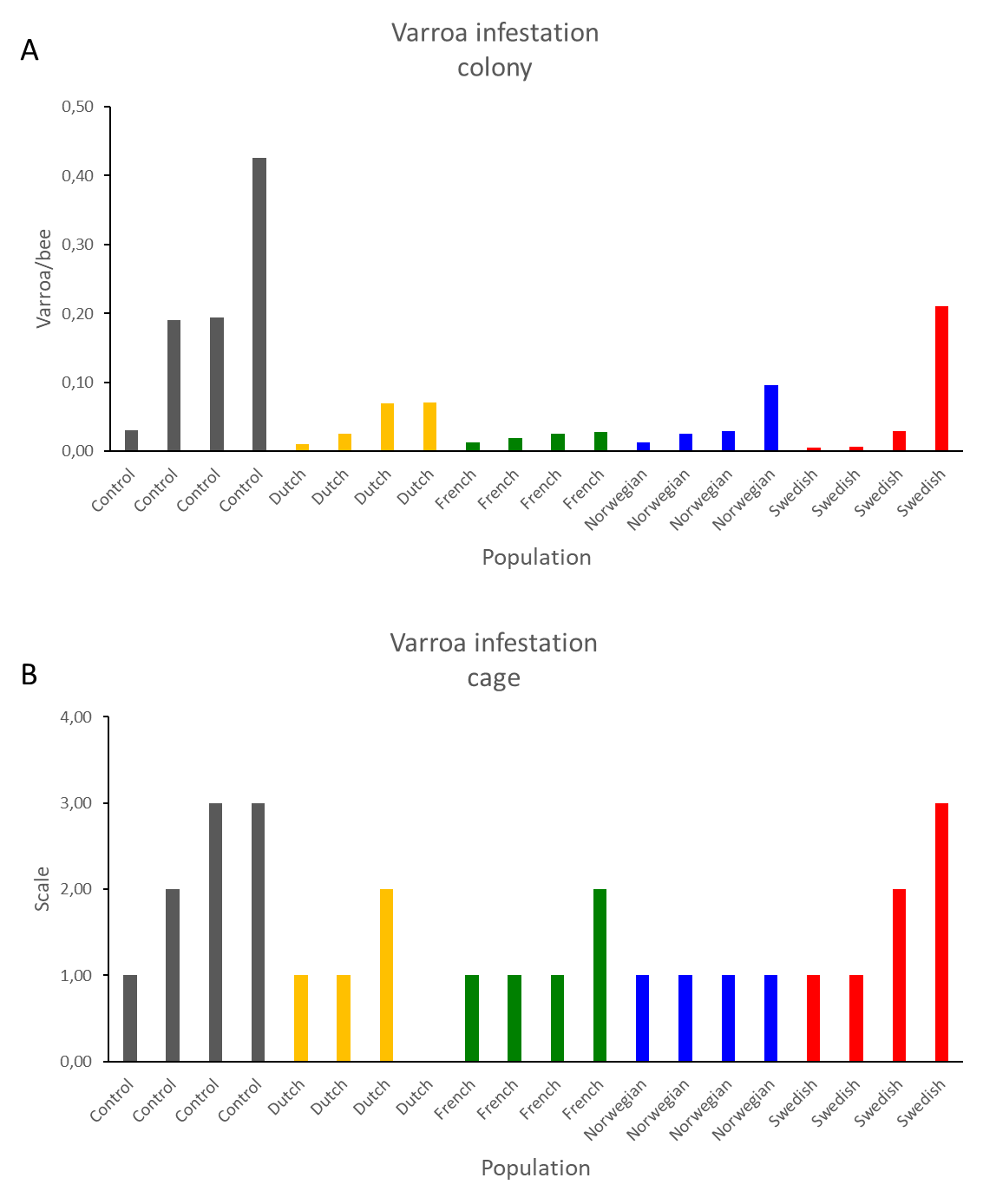


**Supplementary Figure 1.** Colony-level phoretic varroa infestation rates on August 17, 2017 for the colonies used in the adult infection experiments (Supplementary Figure 1a) and subjective assessment on a 0-3 scale of the varroa infestation of the emerged adult bees from the same colonies (Supplementary Figure 1b).


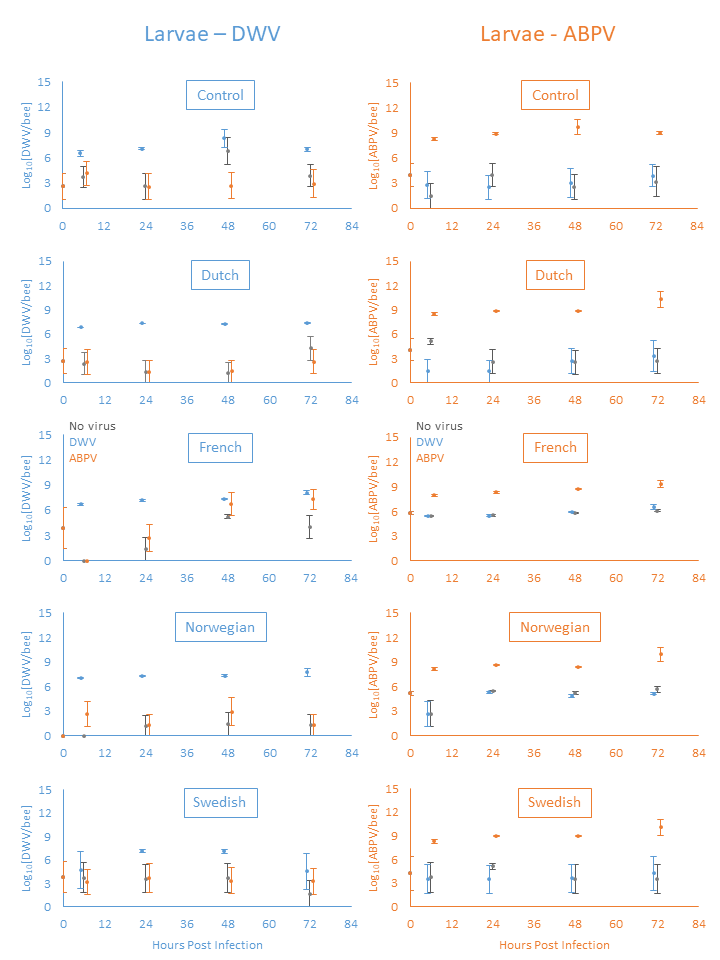

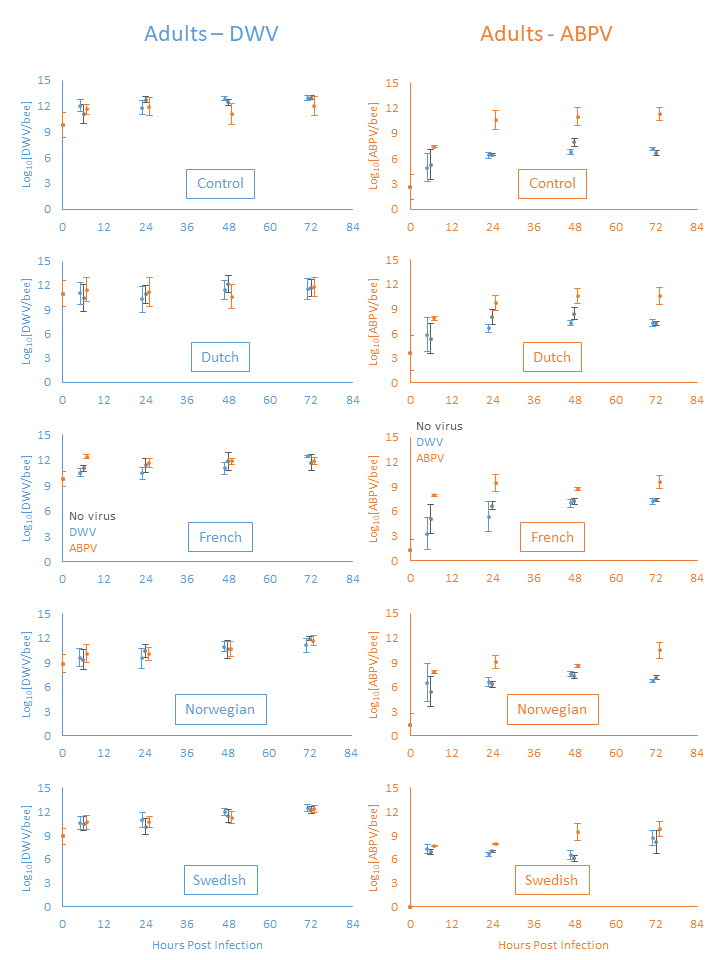


**Supplementary Figure 2.** Graphical representation of the raw data for the larval (left panel) and adult (right panel) virus infection time-course experiments, showing the average DWV (left-blue) and ABPV (right-orange) titres across the infection time-course for DWV-inoculated (blue), ABPV-inoculated (orange) and non-inoculated (grey) bees from four colonies each of the virus-susceptible Control population and the Dutch, French, Norwegian and Swedish varroa-resistant populations. Error bars indicate the standard error for each individual time-point based on the four replicate colonies.


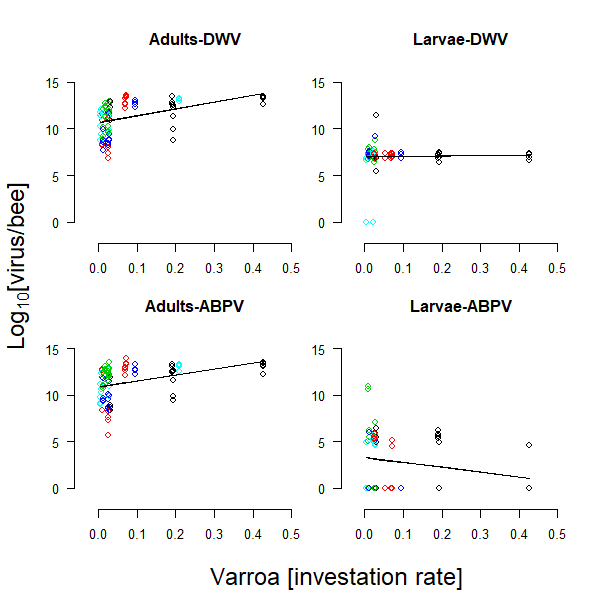


**Supplementary Figure 3.** Relationship between the varroa infestation rate and the titre of the inoculated virus for each of the colonies in the experiment, shown separately for the larval and adult inoculation experiments in adults and larvae. Lines represent linear predictions to illustrate the direction of the effect shown in table 1. The coloured circles show the original data for the Control (black), Dutch (orange), French (green), Norwegian (blue) and Swedish (red) populations.

**Supplementary Table 1.** Results of the Generalized Linear Model analyses of the DWV and ABPV infection time-course studies in larvae and adults. Separate analyses were run for all four combinations of the two different **Life Stage**s (larvae and adults) and the two different response variables; **[DWV] ± SE** titre: blue (Supplementary tables 1a and 1b) and **[ABPV] ± SE** titre: orange (Supplementary tables 1c and 1d). The two explanatory variables analysed were the **Population** of origin (Control Dutch, French, Norwegian or Swedish, the effect of the colony-level phoretic **varroa** infestation rate for each colony, and the type of **Inoculum** used in the infection experiments: DWV (blue), ABPV (orange) or none (grey). The estimated virus titres, standard errors (SE) and degrees of freedom (**DF**) are based on four replicate colonies per population (three for Swedish larvae), each with four post-inoculation sampling time-points (6, 24, 48 & 72 hpi) included as a random factor in the analyses. The significance of the difference between the virus titres was tested with a multiple comparisons Tukey's t-test with adjusted P value, with ***P*** indicating the probability of obtaining this t-value by chance. The final column shows different group allocations of the virus titres with regard to the population of origin and the inoculation treatment.

| **Life Stage** | **Inoculum** | **Population** | **[DWV] ± SE** | **DF** | **t-value** | ***P*** | **group(s)** |
| --- | --- | --- | --- | --- | --- | --- | --- |
| **Larva** | DWV | Control | 7.98 ± 0.81 | 34.19 | 9.90 | <0.001 | g |
| **Larva** | DWV | Dutch | 6.78 ± 0.74 | 25.49 | 9.22 | <0.001 | efg |
| **Larva** | DWV | French | 7.19 ± 0.75 | 26.92 | 9.53 | <0.001 | fg |
| **Larva** | DWV | Norwegian | 7.40 ± 0.73 | 26.10 | 10.15 | <0.001 | fg |
| **Larva** | DWV | Swedish | 5.65 ± 0.82 | 37.71 | 6.93 | <0.001 | dg |
| **Larva** | ABPV | Control | 3.85 ± 0.81 | 34.19 | 4.78 | <0.001 | ade |
| **Larva** | ABPV | Dutch | 1.57 ± 0.74 | 25.49 | 2.14 | 0.042 | ab |
| **Larva** | ABPV | French | 4.06 ± 0.75 | 26.92 | 5.39 | <0.001 | bcde |
| **Larva** | ABPV | Norwegian | 2.10 ± 0.73 | 26.10 | 2.88 | 0.008 | ac |
| **Larva** | ABPV | Swedish | 3.14 ± 0.82 | 37.71 | 3.85 | <0.001 | ad |
| **Larva** | none | Control | 4.69 ± 0.76 | 27.61 | 6.19 | <0.001 | cdf |
| **Larva** | none | Dutch | 1.98 ± 0.68 | 19.45 | 2.89 | 0.009 | ac |
| **Larva** | none | French | 2.78 ± 0.70 | 20.86 | 3.96 | <0.001 | ad |
| **Larva** | none | Norwegian | 0.84 ± 0.67 | 19.82 | 1.24 | 0.230 | a |
| **Larva** | none | Swedish | 3.07 ± 0.75 | 28.54 | 4.08 | <0.001 | ad |
|  |  |  |  |  |  |  |  |
| **Life Stage** | **Inoculum** | **Population** | **[DWV] ± SE** | **DF** | **t-value** | ***P*** | **group(s)** |
| **Adult** | DWV | Control | 10.96 ± 0.66 | 14.31 | 16.63 | <0.001 | ab |
| **Adult** | DWV | Dutch | 11.19 ± 0.64 | 12.54 | 17.62 | <0.001 | ab |
| **Adult** | DWV | French | 11.52 ± 0.64 | 12.59 | 18.09 | <0.001 | ab |
| **Adult** | DWV | Norwegian | 10.03 ± 0.64 | 12.70 | 15.74 | <0.001 | a |
| **Adult** | DWV | Swedish | 10.96 ± 0.64 | 13.21 | 17.01 | <0.001 | ab |
| **Adult** | ABPV | Control | 10.46 ± 0.66 | 14.16 | 15.92 | <0.001 | a |
| **Adult** | ABPV | Dutch | 11.33 ± 0.64 | 12.54 | 17.84 | <0.001 | ab |
| **Adult** | ABPV | French | 12.34 ± 0.64 | 12.59 | 19.37 | <0.001 | b |
| **Adult** | ABPV | Norwegian | 10.44 ± 0.64 | 13.18 | 16.22 | <0.001 | a |
| **Adult** | ABPV | Swedish | 10.74 ± 0.64 | 13.21 | 16.67 | <0.001 | ab |
| **Adult** | none | Control | 10.85 ± 0.65 | 13.29 | 16.81 | <0.001 | ab |
| **Adult** | none | Dutch | 11.60 ± 0.62 | 11.17 | 18.82 | <0.001 | ab |
| **Adult** | none | French | 11.65 ± 0.62 | 11.61 | 18.69 | <0.001 | ab |
| **Adult** | none | Norwegian | 10.33 ± 0.62 | 11.32 | 16.70 | <0.001 | a |
| **Adult** | none | Swedish | 10.43 ± 0.62 | 11.35 | 16.84 | <0.001 | a |
|  |  |  |  |  |  |  |  |
| **Life Stage** | **Inoculum** | **Population** | **[ABPV] ± SE** | **DF** | **t-value** | ***P*** | **group(s)** |
| **Larva** | DWV | Control | 3.28 ± 0.68 | 21.30 | 4.83 | <0.001 | ab |
| **Larva** | DWV | Dutch | 1.74 ± 0.63 | 16.14 | 2.76 | 0.014 | a |
| **Larva** | DWV | French | 5.39 ± 0.64 | 17.27 | 8.38 | <0.001 | b |
| **Larva** | DWV | Norwegian | 4.62 ± 0.62 | 15.97 | 7.41 | <0.001 | bc |
| **Larva** | DWV | Swedish | 3.34 ± 0.69 | 22.53 | 4.87 | <0.001 | ab |
| **Larva** | ABPV | Control | 9.19 ± 0.68 | 21.30 | 13.51 | <0.001 | d |
| **Larva** | ABPV | Dutch | 8.66 ± 0.63 | 16.14 | 13.76 | <0.001 | d |
| **Larva** | ABPV | French | 8.14 ± 0.64 | 17.27 | 12.65 | <0.001 | d |
| **Larva** | ABPV | Norwegian | 8.93 ± 0.62 | 15.97 | 14.32 | <0.001 | d |
| **Larva** | ABPV | Swedish | 8.67 ± 0.69 | 22.53 | 12.64 | <0.001 | d |
| **Larva** | none | Control | 3.21 ± 0.65 | 17.56 | 4.98 | <0.001 | ab |
| **Larva** | none | Dutch | 2.90 ± 0.59 | 12.80 | 4.89 | <0.001 | ac |
| **Larva** | none | French | 5.26 ± 0.61 | 13.85 | 8.68 | <0.001 | b |
| **Larva** | none | Norwegian | 4.94 ± 0.59 | 12.60 | 8.44 | <0.001 | b |
| **Larva** | none | Swedish | 3.62 ± 0.64 | 17.50 | 5.65 | <0.001 | ab |
|  |  |  |  |  |  |  |  |
| **Life Stage** | **Inoculum** | **Population** | **[ABPV] ± SE** | **DF** | **t-value** | ***P*** | **group(s)** |
| **Adult** | DWV | Control | 4.83 ± 1.18 | 6.08 | 4.11 | 0.006 | b |
| **Adult** | DWV | Dutch | 5.86 ± 1.16 | 5.72 | 5.06 | 0.003 | bd |
| **Adult** | DWV | French | 5.19 ± 1.16 | 5.72 | 4.47 | 0.005 | b |
| **Adult** | DWV | Norwegian | 5.99 ± 1.16 | 5.75 | 5.16 | 0.002 | bd |
| **Adult** | DWV | Swedish | 6.41 ± 1.17 | 5.85 | 5.50 | 0.002 | bcd |
| **Adult** | ABPV | Control | 9.06 ± 1.18 | 6.04 | 7.71 | <0.001 | e |
| **Adult** | ABPV | Dutch | 9.04 ± 1.16 | 5.72 | 7.80 | <0.001 | e |
| **Adult** | ABPV | French | 8.56 ± 1.16 | 5.72 | 7.39 | <0.001 | ce |
| **Adult** | ABPV | Norwegian | 8.00 ± 1.17 | 5.86 | 6.86 | <0.001 | de |
| **Adult** | ABPV | Swedish | 7.83 ± 1.17 | 5.85 | 6.71 | <0.001 | ade |
| **Adult** | none | Control | 5.17 ± 1.16 | 5.84 | 4.43 | 0.005 | b |
| **Adult** | none | Dutch | 6.70 ± 1.14 | 5.39 | 5.87 | 0.002 | bcd |
| **Adult** | none | French | 5.66 ± 1.15 | 5.48 | 4.94 | 0.003 | ab |
| **Adult** | none | Norwegian | 5.52 ± 1.14 | 5.42 | 4.83 | 0.004 | b |
| **Adult** | none | Swedish | 5.73 ± 1.14 | 5.42 | 5.01 | 0.003 | ab |

**Supplementary Table 2.** Model performance parameters for the Cox Proportional Hazard analyses of the adult bee mortality data for DWV-inoculated, ABPV-inoculated and non-inoculated bees. Each bee in each cage at each time-check constituted an **observation**, which continued until the bee died (**Number of deaths**). Shown are the number of **iterations** required for the models to converge; the **variance of the random effects** (apiary location); the **concordance** of the data and the ability of the model to explain the data, as determined through a **Likelihood ratio test**, with the associated **Degrees of Freedom** and the **Probability** of obtaining the observed **I-likelihood** value by chance.

| **Inoculum** | **DWV** | **ABPV** | **none** |
| --- | --- | --- | --- |
| Number of observations until death | 3263 | 3221 | 3502 |
| Number of deaths | 250 | 233 | 97 |
| Iterations: outer | 14 | 11 | 12 |
| Iterations: Newton-Raphson | 35 | 28 | 31 |
| Variance of random effect | 0.788 | 0.864 | 0.560 |
| Concordance | 0.741 | 0.803 | 0.710 |
| I-likelihood | -1462.5 | -1304.3 | -588.7 |
| Likelihood ratio test | 169.4 | 285.5 | 60.36 |
| Degrees of Freedom | 7.88 | 7.87 | 7.62 |
| Probability | <2x10^-16^ | <2x10^-16^ | <3x10^-10^ |
